# Carcinoma involving the gallbladder: a retrospective review of 23 cases - pitfalls in diagnosis of gallbladder carcinoma

**DOI:** 10.1186/1746-1596-7-10

**Published:** 2012-01-27

**Authors:** Tran H Giang, Tran TB Ngoc, Lewis A Hassell

**Affiliations:** 1 Ho Chi Minh City College of Medicine and Pharmacy, Ho Chi Minh City, Vietnam; 2 University of Oklahoma Health Sciences Center, Oklahoma City, Oklahoma

**Keywords:** Carcinoma, gallbladder neoplasm, immunohistochemistry, histology, misdiagnosis

## Abstract

**Background:**

Carcinoma of the gallbladder (GBC) clinically mimics benign gallbladder diseases and often escapes detection until advanced stage. Despite the frequency of cholecystectomy, diagnosis of GBC remains problematic in many situations. We sought to identify pathologic features that contribute to the difficulty in recognition of GBC.

**Methods:**

We identified 23 patients (ranged from 45 to 86 years, male to female ratio 1:4.5) with carcinoma involving the gallbladder referred to an academic medical center over a period of 10 years for study. This includes 10 cases of primary GBC, 6 cases of metastatic tumor to gallbladder, 6 cases of directly invasive adenocarcinoma arising elsewhere in the biliary tree, and one case of unidentified origin adenocarcinoma. Primary tumors include adenocarcinoma not otherwise specified (NOS) in 6 cases, papillary adenocarcinoma in 2 cases, and single cases of undifferentiated carcinoma and combined adenocarcinoma and neuroendocrine carcinoma (NEC). Metastatic tumors to gallbladder were from a wide range of primary sites, predominantly the gastrointestinal tract.

**Results:**

These cases illustrate seven potential pitfalls which can be encountered. These include: 1) mistakenly making a diagnosis of adenocarcinoma of gallbladder when only benign lesions such as deeply penetrating Rokitansky-Aschoff sinuses are present (overdiagnosis), 2) misdiagnosing well-differentiated invasive carcinoma with minimal disease as benign disease (underdiagnosis), 3) differentiating between primary NEC of gallbladder and metastasis, 4) confusing primary mucinous adenocarcinoma of gallbladder with pseudomyxoma peritonei from a low grade appendiceal neoplasm disseminated to gallbladder, 5) confusing gangrenous necrosis related to cholecystitis with geographic tumoral necrosis, 6) undersampling early, grossly occult disease, and 7) misinterpreting extracellular mucin pools.

**Conclusions:**

Clinical history and a high index of suspicion are prerequisite to detecting GBC. Detection of GBC at an early stage is difficult because the symptoms mimic benign gallbladder diseases. Misinterpretation of subtle microscopic abnormalities contributes diagnostic failures in early cases. Careful attention to any evidence of mural thickening, thorough sampling, particularly in older patients, and close examination of any deeply situated glandular structures are critical. Correlations with radiographic and clinical findings are important helps to avoid misdiagnosis in this commonly resected organ.

## Background

Gallbladder carcinoma (GBC) is a relatively uncommon neoplasm that shows female predominance (female to male ratio, 3-4: 1), possibly related to the increased incidence of calculi in women. The mean age of patients is 65 years, compared to a mean age of presentation with cholelithiasis of 49 years. In the United States, Hispanic and Native Americans have a higher rate of gallbladder cancer than other ethnic groups. Gallbladder carcinomas are associated with gallstones (80%), porcelain gallbladder (10-20%), and abnormal choledochopancreatic duct junction. Size of the gallstones may also be a risk factor, as patients with stones larger than 3 cm have a significantly greater risk of developing carcinoma. Recently, clinical and epidemiological studies have suggested a link between gallstone disease [1], GBC as well as other hepatobiliary diseases and previous infection with *Helicobacter *species [2]. Sixty percent of GBC arise in the fundus. Invasion of liver, lymph nodes and other organs are frequent. Histologically, most GBC are pancreatobiliary-type adenocarcinomas, showing variable degrees of differentiation. Some arise in association with a noninvasive papillary neoplasm. Additional, several histologic variants of adenocarcinoma are recognized: papillary, intestinal, mucinous, signet-ring cell and clear cell. Many tumors contain more than one histologic variant. The remaining epithelial cell types occurring in the gallbladder include adenosquamous carcinoma, squamous cell carcinoma, small cell carcinoma, and undifferentiated carcinoma. The determination of the histological type of the tumor and differential diagnosis from gallbladder adenocarcinoma are often difficult [3,4]. Failure to detect early disease contributes to a generally poor prognosis. Preliminary observations indicating potentially frequent under- and over-diagnosis of GBC led us to undertake this study. In the present report, we review our experience with GBC over a 10 year period, noting some of the pitfalls which can be encountered. We also suggest some ways whereby these pitfalls may be avoided.

## Methods

This retrospective study was carried out from data on 23 patients with carcinoma of the gallbladder retrieved from the surgical pathology files of an academic medical center between January 2001 and November 2011. Patients with pathologic materials referred to the University of Oklahoma Medical Center (OUMC) and a diagnosis of carcinoma involving the gallbladder were eligible for the study. The surgically resected specimens were fixed in 10% neutral-buffered formalin and embedded in paraffin. Sections were used for hematoxylin and eosin staining and immunohistochemical examinations. Slides from some patients were reviewed as whole slide digital images if the primary materials had been returned to a referring institution after the patient was seen at OUMC.

## Results

Of our 23 cases, 18 presented few difficulties in diagnosis. The most common presenting symptoms of primary GBC were abdominal pain predominantly in the epigastric and right upper quadrant, jaundice, nausea, vomiting, anorexia, and weight loss (50%). Imaging studies performed in all the patients showed the presence of gallstones in 14 (60%) cases overall, and in 90% of cases of primary GBC. Grossly evident tumor was seen on initial pathologic examination in 12 of 23 cases (52%). Eight (80%) of 10 primary GBC cases had tumor masses, while 2 cases grossly presented no visible tumor. The most common tumor sites were in the body and the neck of gallbladder. Carcinoma was suspected pre-operatively in only 5 patients (22%), while the clinical diagnosis in the remainder was acute cholecystitis, stone disease and bile duct tumor. Surgical specimens from the 10 patients with primary adenocarcinoma showed adenocarcinoma NOS in 6 cases, papillary adenocarcinoma in 2 cases, and single cases of undifferentiated carcinoma and combined adenocarcinoma and NEC (See Table 1). In the adenocarcinoma NOS group, 4 cases were moderately differentiated and 2 cases were well differentiated carcinoma.

**Table 1:** Clinical and pathologic features of patients with gallbladder malignancies

Feature of primary GB carcinoma	N_0 _(Percentage)
Clinical symptoms (abdominal pain, jaundice, nausea and vomiting, weight loss)	5/10 (50%)

Mass	8/10 (80%)

Gallstones	9/10 (90%)

Mass associated with gallstones	8/9 (89%)

Pre-op diagnosis benign diseases	5/10 (50%)

Pre-op diagnosis GB cancer	2/10 (20%)

Pre-op diagnosis bile duct tumor	3/10 (30%)

Malignant postoperative diagnosis	Primary: 10/23 (43%)
	
	Metastasis to GB: 6/23 (26%)
	
	Bile duct carcinoma invasive into GB: 6/23 (26%)
	
	Unidentified origin carcinoma: 1/23 (5%)

Five of our cases presented particular challenges in diagnosis. These included one patient in whom the diagnosis was made only after the initial cholecystectomy specimen was reviewed four years later when he presented with bowel obstructive symptoms due to peritoneal carcinomatosis, one patient in whom surface dysplasia involving Rokitansky-Aschoff sinuses (RAS) and adenomyosis was mistaken for deeply invasive carcinoma, one patient in whom geographic tumoral necrosis closely resembled acute gangrenous necrosis more typical in acute cholecystitis, and one patient with isolated mucin pools and only rare tumor cells. One additional patient with a combined adenocarcinoma and NEC presented a challenge in differential diagnosis of primary vs. metastasis as well (See Table 2).

**Table 2:** Summary of case details in problematic cases:

Case		Clinical Presentation	Radiology	Gross Description	Final Diagnosis
A	66 yo male	abdominal pain, 4 years post cholecystectomy for ruptured cholecystitis and cholelithiasis	CT: peritoneal carcinomatosis and free fluid	Peritoneal implants with slight granular thickening of peritoneum Gallbladder wall 5 mm thick no mass, and gallstones in the lumen	Well differentiated adenocarcinoma of the gallbladder and peritoneal metastasis

B	49 yo female	On routine examination, pelvic mass and gallbladder polyp were found with no remarkable clinical symptoms	CT: an enhancing 2 cm mass in the GB fundus. US: heterogeneous mass with associated internal flow worrisome for GB malignancy: cholelithiasis	Polypoid mass protruding from a stalk (4.0 × 2.3 × 1.4 cm) gallstones. On cut section the polyp was white tan, homogeneous throughout	Combined adenocarcinoma and neuroendocrine carcinoma, poorly differentiated

C	86 yo female	Three days of RUQ pain, nausea, fever. History of breast cancer S/P mastectomy 7 years ago, treated with Tamoxifen, squamous cell carcinoma of nose, ovarian cyst removal	US & CT: gallstones and sludge; pancreatic head mass	BG 9.5 × 6.5 × 6.0 cm. seven firm yellow to black multi-faceted calculi. GB wall thickened to 1.5 cm. Mucosa lined by friable pale tan necrotic tissue extruded into lumen. 3.5 cm maximal tumor size, circumferential at neck and filling up to 30% of the luminal volume	Pancreatic biopsy: adenocarcinoma with abundant lymphocytes. GB: Undifferentiated carcinoma of GB with extensive necrosis and acute/chronic inflammation

D	53 yo female	Increasing abdominal girth over several months	CT & US: moderate volume ascites, 18.5 cm complex right pelvic mass; GB: 1.8 cm polyp	A 5 cm appendix filled with yellow-tan mucoid material. Gallbladder was 7.3 × 3 cm in greatest dimensions with a flesh colored lesion presenting in the serosa. The mucosa was unremarkable	Low grade mucinous neoplasm/adenocarcinoma of the appendix with peritoneal spread

E	45 yo female	RUQ pain	Cholelithiasis	Mural thickness 5 mm; no mass; stones up to 2.5 cm	Focally invasive adenocarcinoma; extensive CIS involving RAS

Abbreviations: GB = gallbladder; CT = computed tomography scan; US = ultrasound examination; RAS = Rokitansky-Aschoff sinuses; RUQ = right upper quadrant; CIS = carcinoma in situ; S/P = status-post

## Discussion

**Figure 1:**
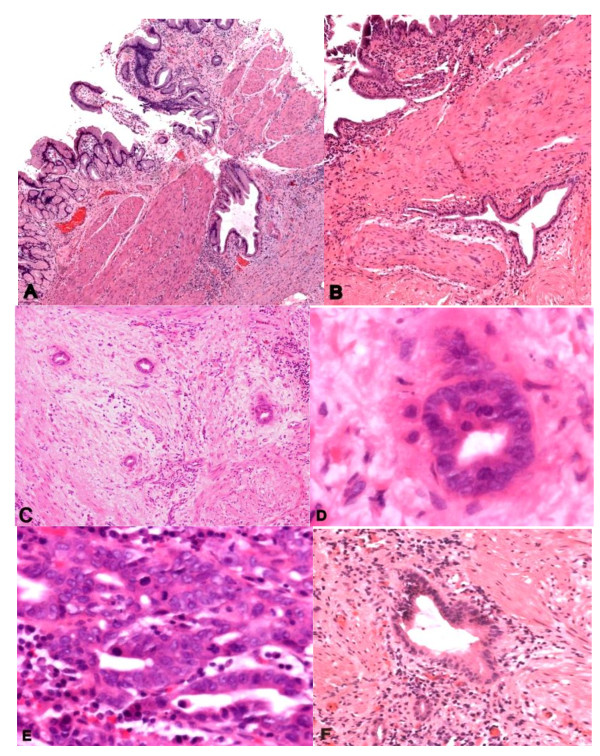
**Benign mimicking malignant, and malignant mimicking benign in the gallbladder**. A-B: RAS extending transmurally, without any associated dysplasia, C: Glands embedded in bundles of smooth muscle resembling RAS, D: Well-differentiated glandular structures embedded deeply in the gallbladder wall with subtle atypia, E: Foci of back to back arrangement of glands lined by layers of highly atypical columnar cell. Many mitoses presented, F: Diffuse wall thickening with intramural diverticula within the wall, mimicking well-differentiated adenocarcinoma of the gallbladder (H&E stain).

Well-differentiated adenocarcinoma of the gallbladder can be difficult to distinguish from RAS, which can be located throughout the gallbladder wall, even extending into perimuscular adipose tissue. RAS are normally continuous, showing a perpendicular orientation to the surface, and typically have undulating, smooth contours (See Figure 1A, B). In contrast, adenocarcinomas show small and variably sized glands with angulated contours [5]. The malignant glands are usually densely packed and may be oriented parallel to the surface. Desmoplasia favors a diagnosis of carcinoma. However, a stromal desmoplastic-like reaction surrounding RAS is not uncommon, especially when there is active cholecystitis. Moreover, cytologic atypia, mitoses, and intraglandular necrosis are all features that favor a diagnosis of adenocarcinoma over benign RAS [4,6].

Two of our cases (see Table 2, A and E) illustrate the pitfalls associated with this challenging aspect of gallbladder microanatomy. Surface dysplasia was identified on initial evaluation of case E and subsequently submitted additional sections demonstrated numerous areas in which dysplasia extended into deeply situated RAS, mimicking transmural invasion of carcinoma. However, some lateral intramural growth was also present without typical associated mucosal stroma in which atypical small glands were closely juxtaposed to smooth muscle. In contrast, another case in our series (case A) demonstrated small glands embedded in bundles of smooth muscle and surrounding a deep vessel, but resembling RAS (See Figure 1C, D). The surface mucosa was inflamed and also associated with some atypia, making it difficult to differentiate from reactive atypia involving the surface and RAS. However, the lining cells of deep glands presented cytological and subtle architectural atypia without significant inflammation. The surface epithelium exhibited multiple areas with high grade dysplasia (See Figure 1D, E). Further, the stroma surrounding the deeply situated glands was more desmoplastic than inflammatory in nature. The morphological diagnosis at the time was cholecystitis. Unfortunately, 4 years post-cholecystectomy, the patient presented with abdominal pain and radiographic evidence of disseminated peritoneal implants. Surgical sampling at this point led to a diagnosis of metastatic well differentiated adenocarcinoma. Immunostains showed the tumor cells to be diffusely positive for CK7 and negative for CK20, WT1, D240, calretinin, CK5/6 and TTF1. MUC-1, MUC-5A, MLUC6, P53 were positive in the tumor cells. Subsequent review of the previously removed gallbladder revealed the small primary tumor and associated surface dysplasia.

Adenomyosis can also be confused with adenocarcinoma of gallbladder. It is a hyperplastic condition characterized by excessive proliferation of surface epithelium with deepened invaginations or diverticula extending into the thickened muscular layer of gallbladder wall, again mimicking well-differentiated adenocarcinoma of the gallbladder. However, the glands in adenomyosis are usually bland cytologically; they show cystic dilatation, and they communicate with the main gallbladder lumen [7,8] (See Figure 1F). Pathologists should be aware of the presence of glandular structures embedded in the gallbladder wall. This condition does not simply suggest RAS or adenomyosis. The precise evaluation of the appearance of the whole lesion may be useful in distinguishing these diseases.

The immunoprofile of GBC is similar to that of bile duct carcinoma (intrahepatic and extrahepatic) and pancreatic carcinoma. Cytokeratin 7 (CK7) is almost always positive, Cytokeratin 20 (CK20) can be positive, more often in extrahepatic bile duct carcinoma than intrahepatic cholangiocarcinoma. In addition, carcinoembryonic antigen-monoclonal (CEA-M), carbohydrate antigen (CA19-9), B72.3, MUC1, and MUC5AC are also positively expressed in bile ducts and GBC but can be focal. MUC overexpression rates are reportedly higher in GBC than in cholecystitis and gallbladder adenoma [7,8].

**Figure 2:**
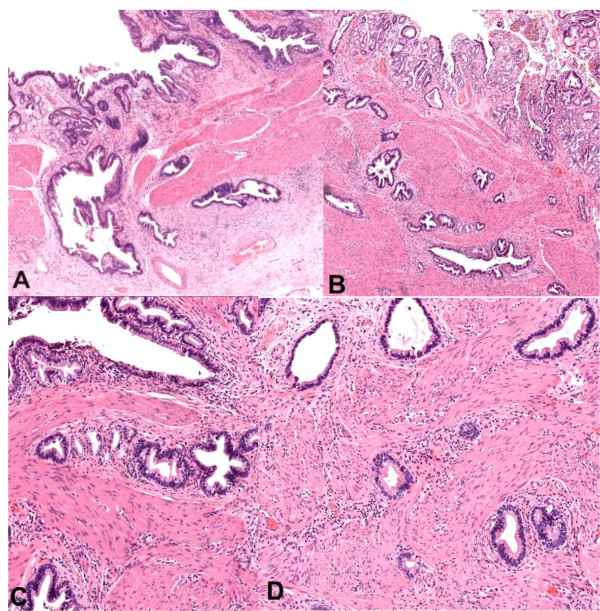
**Dysplasia in RAS and adenomyosis can mimic invasive GBC**. A-B: Dysplastic epithelium within RAS penetrating slightly beyond the wall, C: dysplastic epithelium associated with adenomyosis, D: Foci of intramural invasive carcinoma (H&E stain).

Another potential problem area that should be considered is carcinoma arising from RAS, without tumor mass [3]. We believe this is extremely rare, although conceivably more advanced cancers could have arisen from such a location. Demonstration of this requires a minute adenocarcinoma arising from RAS and located in the wall or subserosa, with no apparent connection to mucosa as in case E above (See Table 2) of a 45-year-old woman with preoperative diagnosis of cholelithiasis. The morphological diagnosis at another hospital was transmurally invasive moderately differentiated adenocarcinoma, though no mass was identifiable grossly and the wall only focally thickened to 5 mm. Subsequent review of the previously removed gallbladder revealed extensive surface dysplasia, with high grade dysplastic epithelium within RAS penetrating slightly beyond the wall (See Figure 2A) and foci of intramural invasive carcinoma (See Figure 2B). The gradual transition between adenocarcinoma cells and RAS with dysplasia was recognized. A key differentiating feature from adenomyosis is that muscular hypertrophy was not pronounced. Cytologic atypia sufficient for a diagnosis of adenocarcinoma should also be evident. In general, carcinoma arising from RAS is small and has a relatively good prognosis. Therefore, careful examination of resected gallbladders is necessary [6], particularly any areas of focal mural thickening.

**Figure 3:**
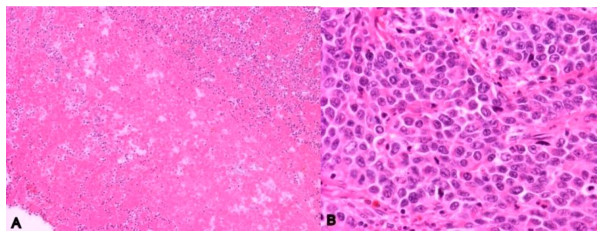
**Geographic necrosis can mask underlying GBC**. A: Extensive geographic necrosis and prominent acute inflammation (H&E stain, X100), B: The tumor was characterized by vesicular nuclei, prominent nucleoli, and scant eosinophilic cytoplasm (H&E stain, X400).

Some important pathologic findings overlap in benign and malignant lesions and may contribute to possible confusion, such as necrosis or extracellular mucus. An acute cholecystitis with parietal necrosis can be confused with an aggressive neoplastic process. Similarly, extensive tumor necrosis with minimal residual viable tumor may mimic acute gangrenous cholecystitis. Almost all cellular detail is lost in necrosis and most immunoperoxidase stains either fail or give misleading, non-specific falsly positive results. The presence of associated clinical signs can be of value to make the diagnosis of carcinoma or acute cholecystitis. The characteristics of the histologic changes in acute cholecystitis such as edema, vascular congestion, hemorrhage, fibrin deposition in the adventitia and adjacent muscle should be noted. Mucosal and mural necrosis may be seen with neutrophils [4,9]. Likewise, thorough histologic sampling is critical in cases with extensive necrosis to reveal diagnostic viable tumor. Since necrosis can occur in acute cholecystitis or a malignant tumor, it is important to adequately sample the gallbladder, including areas without necrosis. This is illustrated by case C above, a case of an 86-year-old woman with preoperative diagnosis of acute suppurative cholecystitis with cholelithiasis. She underwent cholecystectomy. Although gross tumor was present, significant geographic necrosis was present with prominent acute, chronic, and xanthogranulomatous inflammation, and viable tumor was only present in a subset of the well-sampled tumor (See Figure 3).

The finding of focal high grade glandular dysplasia or intramucosal adenocarcinoma within the gallbladder strongly favoured origin of this carcinoma within the gallbladder, despite the history of two other malignancies. IHC stains showed focal moderate tumor immunoreactivity for synaptophysin consistent with focal neuroendocrine differentiation. In this case, her find needle biopsy of the head of the pancreas revealed findings suggestive of typical adenocarcinoma. Given her history of bilateral breast cancer and a pancreatic tumor, metastatic tumor would have been more likely than a primary GBC. Taking a very careful cancer history and maintaining a high index of suspicion together with comparing her current findings with prior histologic appearances and appropriate IHC study was prerequisite to accurate diagnosis. Necrosis may be present in many diseases. An incorrect diagnosis could be made if primarily based on necrosis.

**Figure 4:**
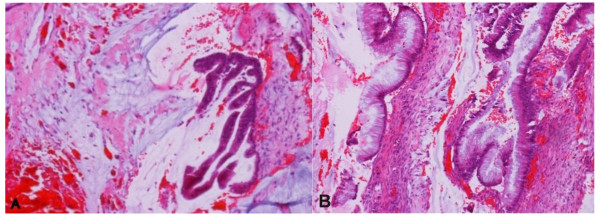
**Extracellular mucin can originate from mucinous metaplasia, GBC or metastasis**. A: Expansile pools of mucin with scattered glands, the mucinous epithelium exhibited cytologic atypia (H&E stain, X100), B: Neoplastic glands within the wall of gallbladder (H&E stain, X100).

Another potentially confusing factor is extracellular mucin. Mucinous carcinoma of gallbladder is uncommon, representing only 4% of all malignancies. It is characterized by small clusters of malignant epithelial cells surrounded by large deposits of extracellular mucin. In general, there are few epithelial glandular elements and when present, they are often distended with mucin [4]. In case D above (See Table 2), radiographic study suggested the possibility of a primary gallbladder neoplasm or a gynecologic neoplasm. At exploration, extensive peritoneal mucinous tumor was present which was removed as much as possible, along with the appendix, gallbladder, and pelvic mass. The appendix showed a villiform mucinous epithelial proliferation replacing the normal appendiceal mucosa. The peritoneal and gallbladder samples were characterized by abundant pools of mucin containing scattered single infiltrating glands and cellular proliferations lined by mucinous epithelium with moderate to occasional high grade cytologic atypia (See Figure 4). The mucinous epithelium in the peritoneal lesions and gallbladder were the same as in the appendiceal lesion.

A diagnosis of mucinous carcinoma should be suspected from gross examination when large amounts of mucus are found in the primary tumor. However, it was easily mistaken for pseudomyxoma peritonei disseminated to gallbladder. Pseudomyxoma peritonei (PMP) is an uncommon disease characterized by abundant extracellular mucin in the peritoneum. It is an older, broad descriptive term embracing a wide spectrum of biological behaviour of neoplasms from the benign and borderline to malignant lesions. PMP is usually associated with a mucinous neoplasm in the appendix that demonstrates fairly bland well differentiated mucinous epithelium often with minimal nuclear features of malignancy and minimal or no invasion [10,11].

Hence, it can be diagnostically challenging to recognize a primary mucinous tumor of the gallbladder vs. PMP disseminated to gallbladder, especially in cases in which only the gallbladder is removed. Similarly, mucinous metaplasia in the gallbladder may be associated with mucosal ulceration, particularly if stones are present, presenting the appearance of possible pools of surface or submucosal mucin. This circumstance warrants close sectioning to avoid missing a primary mucinous neoplasm of the gallbladder.

**Figure 5:**
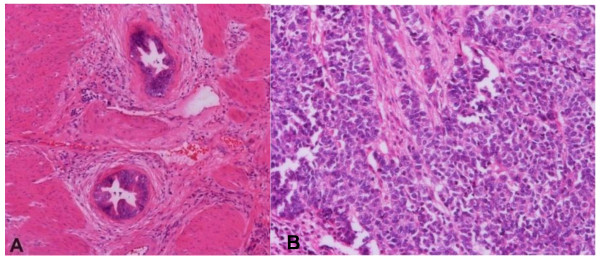
**Mixed types of GBC present difficulty with differential diagnosis**. A: Gallbladder tumor showing well-differentiated tubular adenocarcinoma in the muscle (H&E stain, X100), B: A monotonous proliferation of small round cells with hyperchromatic nuclei and scant cytoplasm, resembling NEC (H&E stain, X200).

Besides pure adenocarcinoma of the gallbladder, many tumors contain more than one histologic variant. In this study, one case was diagnosed as a mixed endocrine-exocrine carcinoma. NET are thought to be derived from enterochromaffin or Kulchitsky cells, which are widely distributed in the body. Consequently, NET can be found in any location in the body, although the sites most commonly affected are the gastrointestinal and bronchopulmonary tracts, representing approximately 67% and 25% of cases, respectively. NET of the gallbladder is extremely rare because normal gallbladder mucosa does not contain neuroendocrine cells. Neuroendocrine cells can be detected at sites of intestinal metaplasia induced by chronic inflammation, which may be the initial step in the development of NET. This NET component may thus be in the deep mucosa of the gallbladder, and potentially produce a more deeply infiltrating tumor involving the serosa and adjacent liver before or without any surface component. Therefore, the differential diagnosis of a primary NEC of the gallbladder and one arising from metastasis is difficult [12-14]. Our case showed a tumorous lesion involving the gallbladder wall accompanied by atypical hyperplastic and dysplastic epithelium. Although the predominant element in the tumorous lesion was adenocarcinoma, there were some foci composed of small cells with neuroendocrine morphology at the microscopic level (See Figure 5).

This patient's immunohistochemistry staining results were positive for cytokeratin and synaptophysin. These findings were suggestive of mixed endocrine-exocrine carcinoma involving full thickness of the gallbladder wall. In summary, NET of the gallbladder is extremely rare. As a single dominant element, metastasis may be more common than primary origin in the gallbladder.

## Conclusion

Carcinoma of the gallbladder is a poor prognosis malignancy since it usually presents at a very advanced stage. Detection of GBC at an earlier stage is very difficult because the symptoms most of the time mimic benign gallbladder diseases like chronic cholecystitis, adenomyosis and because the absence of a gross lesion makes pathologic detection problematic. Further, we have illustrated and identified from our experience other pathologic factors leading to misdiagnosis. Misinterpretation of subtle microscopic abnormalities appears to contribute to improper diagnosis in some of the early cases. Careful attention to any evidence of mural thickening, thorough sampling, particularly in older patients, and close examination of any deeply situated glandular structures that may be mimicking RAS are critical. Further, correlation with radiographic and clinical findings can also be important helps to avoid misdiagnosis in this commonly resected organ.

## Abbreviations

CA19: 9 carbohydrate antigen; CEA-M: carcinoembryonic antigen-monoclonal; CIS: carcinoma in situ; CK7: cytokeratin 7; CK20: cytokeratin 20; CT: computed tomography scan; GB: gallbladder; GBC: carcinoma of the gallbladder; H&E stain: hematoxylin and eosin stain; IHC: immunohistochemistry stains; NEC: neuroendocrine carcinoma; NET: neuroendocrine tumor; NOS: not otherwise specified; PMP: pseudomyxoma peritonei; RAS: Rokitansky:Aschoff sinuses; RUQ: right upper quadrant; S/P: status-post; US: ultrasound examination.

## Competing interests

The authors declare that they have no competing interests relevant to the contents of this submission.

## Authors' contributions

THG reviewed the pathologic materials, case histories, photographed the slides and wrote much of the manuscript. TTBN also reviewed the pathologic materials, retrieved clinical data, and assisted in writing and editing the manuscript. LAH conceived the study, identified suitable patients, reviewed pathologic materials, participated in preparing images and virtual slides, and edited the manuscript. All authors read and approved the final manuscript.
